# *In vivo *silencing of alpha-synuclein using naked siRNA

**DOI:** 10.1186/1750-1326-3-19

**Published:** 2008-11-01

**Authors:** Jada Lewis, Heather Melrose, David Bumcrot, Andrew Hope, Cynthia Zehr, Sarah Lincoln, Adam Braithwaite, Zhen He, Sina Ogholikhan, Kelly Hinkle, Caroline Kent, Ivanka Toudjarska, Klaus Charisse, Ravi Braich, Rajendra K Pandey, Michael Heckman, Demetrius M Maraganore, Julia Crook, Matthew J Farrer

**Affiliations:** 1 Department of Neuroscience, Mayo Clinic, 4500 San Pablo Road, Jacksonville, FL 32224, USA; 2 Alnylam Pharmaceuticals, 300 3rd St, Cambridge, MA 02142, USA; 3 Department of Biostatistics, Mayo Clinic, 4500 San Pablo Road, Jacksonville, FL 32224, USA; 4 Department of Neurology, Mayo Clinic, 200 1st St SW, Rochester, MN 55905, USA; 5 ReNeuron, 10 Nugent Road, Surrey Research Park, Guildford, Surrey, GU2 7AF, UK

## Abstract

**Background:**

Overexpression of α-synuclein (SNCA) in families with multiplication mutations causes parkinsonism and subsequent dementia, characterized by diffuse Lewy Body disease *post-mortem*. Genetic variability in *SNCA *contributes to risk of idiopathic Parkinson's disease (PD), possibly as a result of overexpression. *SNCA *downregulation is therefore a valid therapeutic target for PD.

**Results:**

We have identified human and murine-specific siRNA molecules which reduce *SNCA in vitro*. As a proof of concept, we demonstrate that direct infusion of chemically modified (naked), murine-specific siRNA into the hippocampus significantly reduces *SNCA *levels. Reduction of *SNCA *in the hippocampus and cortex persists for a minimum of 1 week post-infusion with recovery nearing control levels by 3 weeks post-infusion.

**Conclusion:**

We have developed naked gene-specific siRNAs that silence expression of *SNCA in vivo*. This approach may prove beneficial toward our understanding of the endogenous functional equilibrium of *SNCA*, its role in disease, and eventually as a therapeutic strategy for α-synucleinopathies resulting from *SNCA *overexpression.

## Background

The importance of α-synuclein in the pathogenesis of Parkinson's disease (PD) initially emerged in 1997 when Polymeropoulos and colleagues reported that a missense A53T mutation in the α-synuclein gene (*SNCA*) causes familial parkinsonism in four seemingly unrelated kindreds [1]. Subsequently, *SNCA *A30P and E46K missense mutations were found to cause familial Lewy Body parkinsonism [2,3]. The importance of the α-synuclein protein (non-amyloid component precursor; NACP) was confirmed through its recognition as a major component of both Lewy bodies, the pathological hallmark of PD and dementia with Lewy bodies (DLB), and of glial cytoplasmic inclusions in multiple system atrophy (MSA) [4].

In addition to missense mutations, multiplication of the normal *SNCA *locus can cause familial PD. Singleton *et al*. first reported genomic triplication of the *SNCA *locus in affected family members with early onset, parkinsonism, with subsequent cognitive dysfunction [5]. *Post-mortem *exam revealed profound neuronal loss in the *substantia nigra *(SN) and widespread Lewy pathology from the cortex to the basal ganglia [6,7]. Additional *de novo SNCA *duplications and triplications have since been reported in French, Japanese, Korean and Swedish-American families [8-14]. Functionally, *SNCA *multiplications result in a copy-number related increase in both α-synuclein RNA and protein [8,15], and disease onset and severity are associated with gene dosage [11]. Taken together, this provides compelling evidence that *SNCA *overexpression can result in Lewy body parkinsonism and dementia.

Although *SNCA *multiplication remains a rare cause of inherited PD, common genetic variability in the *SNCA *locus is a risk factor for idiopathic PD [16-18]. The effects may be mediated by elevated mRNA/protein expression [8,15,19,20]. Hence therapy aimed at reducing *SNCA *expression levels may provide therapeutic benefit for patients with either familial or idiopathic PD.

The use of double-stranded RNAs for the silencing of genes was first accomplished in nematodes [21]. Molecules of 21 and 22 nucleotides had the most activity in *Drosophila*, and these reagents were named short interfering (si)RNAs [22]. siRNA induces the formation of an RNA-induced silencing complex (RISC) which acts as an endonuclease on target RNA [23], yielding a powerful tool which can be used to reduce expression of specific genes. To exploit the therapeutic potential of siRNA, we developed naked siRNA molecules against *SNCA *that are resistant to endo- and exonuclease activity in serum and yield species-specific reduction of *SNCA in vitro*. We then injected them into the *Cornu Ammonis *(CA1) of the hippocampus of mice and demonstrated a reduction in *SNCA *mRNA levels by quantitative reverse transcription-polymerase chain reaction (RT-PCR) and *in situ *hybridization throughout the hippocampus and cortex. Additionally, we have demonstrated that α-synuclein protein expression in these same cells is qualitatively reduced. This protocol will be invaluable for improving our understanding of the *in vivo *dynamics of *SNCA*, assessing the impact of *SNCA *silencing in the pathogenesis of animal models of PD, and perhaps may hold promise as a future neuroprotective therapy for PD in humans.

## Methods

### *In vitro *characterization of siRNA

Detailed method for *in vitro *studies including siRNA synthesis, cell culture and transfection conditions, fluorescent microscopy, RNA and protein analysis and siRNA serum stability analysis are detailed in the additional files section.

### *In vivo *delivery of siRNA

**Figure 1:**
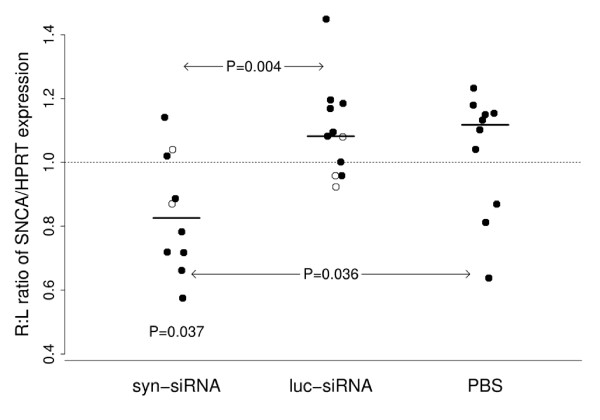
**qRT-PCR analysis of *SNCA *expression following *in vivo *RNAi.***SNCA *siRNA (syn-siRNA), luciferase siRNA (luc-siRNA), or PBS was infused into the right CA1. qRT-PCR was used to determine expression of *SNCA *following RNAi in treated right side compared to the untreated contralateral side (R:L ratio). *SNCA *siRNA had a statistically significant decrease of *SNCA *expression in the right compared to the left side of the brain, and R:L ratios were decreased when compared to controls (vs PBS, p = 0.036; vs. luciferase, p = 0.004). Horizontal lines show medians. Open circles indicate mice in which the cannula was disconnected during treatment or did not function.

**Figure 2:**
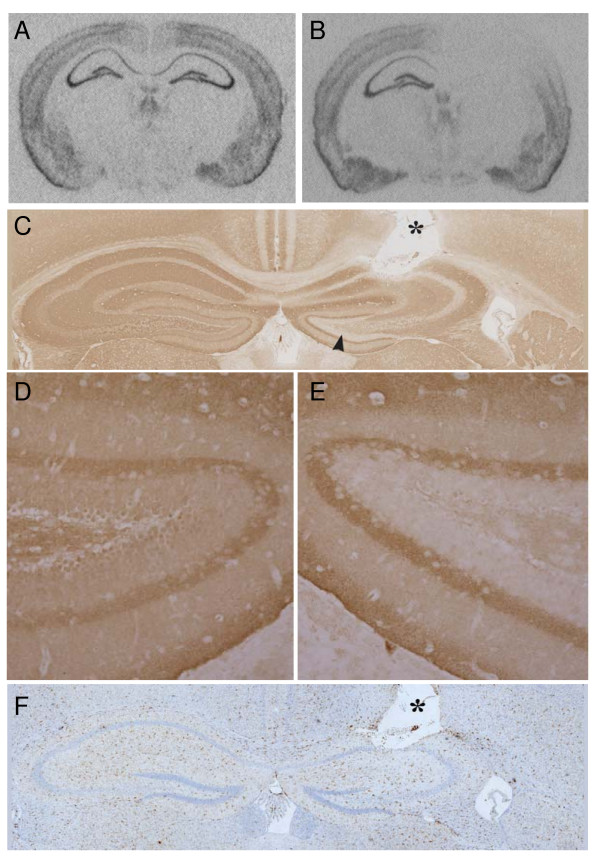
**RNA and protein analysis of α-synuclein expression following *in vivo SNCA *siRNA treatment.** The right CA1 was infused with either PBS, siRNA to luciferase, or siRNA against our *SNCA *target. A typical *SNCA in situ *from an animal treated with (A) PBS or (B) *SNCA *siRNA on the right side compared to the uninjected left sides. While the cannula tract was evident in the right hippocampi of the infused mice (* in C and F), regardless of treatment group, (C) immunostaining for α-synuclein demonstrates considerable knockdown of protein expression (arrowhead) in the hippocampus when the uninjected control side is compared to the *SNCA *siRNA-treated side, also shown in higher magnification (D, E), respectively. (F) Inflammatory changes, as shown by Iba-1 immunostaining for microgliosis, were minimal around the infusion site. Sample brain in (F) showed the highest degree of damage from infusion, in this case from *SNCA *siRNA.

**Figure 3:**
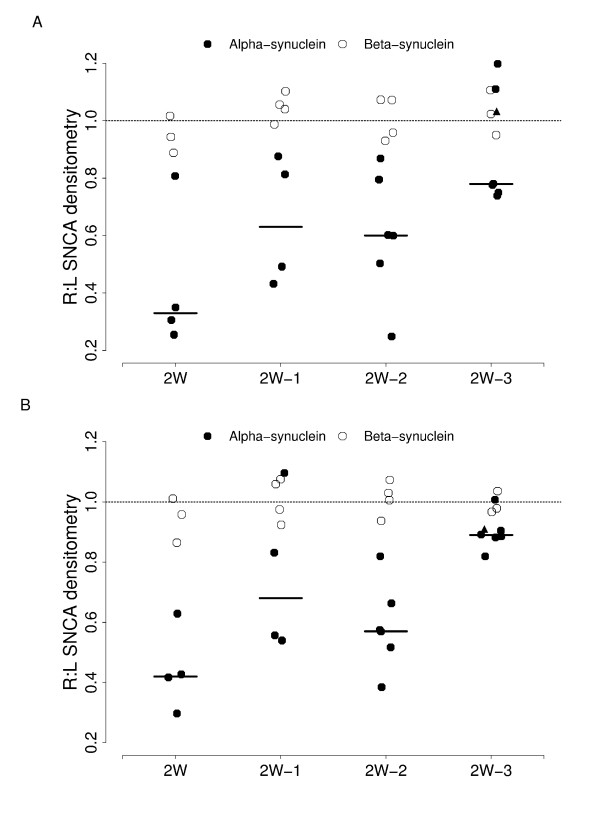
**Silencing of *SNCA *is resilient and target-specific.***SNCA *and *SNCB *expression was assessed by *in situ *hybridization following extended timecourse of *in vivo SNCA *siRNA treatment. Qualitative densitometric analysis was performed on mice treated with *SNCA *siRNA on the right side of the brain and a ratio showing either *SNCA *or *SNCB *expression in the treated (R) and untreated (L) brain was calculated and plotted for each animal within a group. The knockdown of *SNCA *expression (black circle) persists in the (A) CA1 and the (B) cortex two weeks after cannula removal with *SNCA *approaching normal levels by three weeks post-infusion. The closed triangle indicates *SNCA *levels in a mouse in which the cannula was loose at the end of the study. Non-specific silencing of *SNCB *(open circle) was not observed at any timepoint.

All rodent procedures were approved by the Mayo Clinic Institutional Animal Care and Use Committee (IACUC), were in accordance with the National Institute of Health Guide for the Care and Use of Laboratory Animals (NIH Publications No. 80-23, revised 1996) and were performed under the supervision of an institutional veterinarian. Mice were allowed unrestricted access to food and water and were supplemented with gelatin containing (32 mg/ml) acetaminophen from 24 hours prior to surgery until 48 hours post-surgery to minimize pain. The day prior to surgery, Alzet osmotic pumps (model 1002, Durect, Inc., Cupertino, CA) were filled to capacity with siRNA against *SNCA *or luciferase at a concentration of 2 mM in phosphate buffered saline (PBS). Pumps containing only PBS were prepared for an additional control group. Mice were anaesthetized with tribromoethanol (200 mg/kg) and stereotaxic surgery was performed to implant a 3 mm cannula at -1.5 mm posterior and -2.0 mm lateral from bregma and 2.5 mm deep by the insertion of 2 spacers (0.25 mm each) to target the CA1 of the right hippocampus in each 2-month old C57BL/6 female mice [24]. Cannulae were secured to the skull using Loctite adhesive (Durect, Inc., Cupertino, CA). Mice were allowed to recover from the anesthesia under a heat lamp. *SNCA *siRNA, luciferase siRNA or PBS was infused at a rate of 0.25 μl/hour over 15 days using Alzet osmotic pumps attached to 3 mm brain infusion cannulae (Alzet brain infusion kit 3, Durect, Inc., Cupertino, CA; ). Following 15 days infusion, brain tissue was harvested from the 2 week (2 W) cohorts while cannulae were removed on day 15 in cohorts that were allowed to age one week (2 W-1 W), two weeks (2 W-2 W) or three weeks (2 W–3 W) after the cessation of infusion. At harvest, osmotic pumps were recovered. Mice in which the pump had failed or in which the tubing had become disconnected from the pump were included in study groups and in statistical analysis; however, for graphical purposes, the data points for these mice were denoted by distinct symbols when compared to mice in which the pumps were intact and functional. Experiments detailed in Figures 1, 2 and 3 were performed on independent cohorts of mice.

### Taqman qRT-PCR analysis of *SNCA *levels

For Taqman analysis, the hippocampus was rapidly removed from each hemisphere of the brain and snap-frozen on dry ice in separate tubes. In each case, the right hippocampus represented the injected side (*SNCA *siRNA, luciferase siRNA, or PBS) and the left hippocampus was utilized as an untreated control. RNA was extracted by phenol extraction using the TRIzol Reagent (Invitrogen; Carlsbad, CA) with the manufacturer's standard protocol. cDNA was then synthesized using the High Capacity cDNA Archive Kit (Applied Biosystems; Foster City, CA). The following probes were purchased from ABI: GAPDH (Mm99999915_g1), HPRT (Mm01545399_m1), *SNCA *(Mm00447333_m1), and *SNCB *(Mm00504325_m1). Quantitative RT-PCR was performed on an ABI 7900 HT using a 384 well plate with quadruple sample replicates. Results were analyzed using SDS v.2.2 software and the expression data was normalized to mouse *GAPDH *and *HPRT*. Resulting graphs and data were generated using GraphPad Prism v.4 software (GraphPad Software Inc., La Jolla, CA).

### *In situ *analysis of *SNCA *and *SNCB *levels

To ensure sampling consistency, the brain was placed in a tissue matrix and the region anterior and posterior to the hippocampus was removed using a flat blade. The resulting three brain segments were snap frozen on dry ice and stored at -80°C until use. 15 μm thick frozen sections were cut on a cryostat at -18°C throughout the entire hippocampus and air dried for 20 minutes before freezing at -80°C. Frozen sections were removed on dry ice and dried quickly on a slide warmer at 55°C, fixed in 4% paraformaldehyde in 0.1 M Sorensen's Phosphate buffer for 20 minutes, washed twice in PBS and dehydrated in ascending alcohols. Hybridization was performed at 37°C overnight in a moist chamber, with approximately 0.02 ng of [α-^33^P] dATP (Perkin Elmer, Waltham, MA, USA) 3' end labeled probe per 1 μl of hybridization buffer (4× sodium chloride/sodium citrate (SSC), 1× Denhardt's solution, 50% (w/v) de-ionised formamide, 10% (w/v) dextran sulphate, 200 mg/μl herring sperm DNA). The *SNCA *probe (5'GGTCTTCTCAGCCACTGTTGTCACTCCATGAACCAC'3) was designed to exon 3. The *beta-synuclein *(*SNCB) *probe was designed to the 3' untranslated region (UTR) (5'CAGACAGATTGGCTTTATTCATGGACACACTGGG'3). Specific activity of the probe was at least 1 × 10^8 ^counts per minute (cpm)/μg, and after dilution in hybridization buffer corresponded to ~1 × 10^4 ^cpm/μl. Control hybridizations contained a 50-fold molar excess of unlabelled probe to determine non-specific signal. Slides were washed in 1× SSC at room temperature (RT) to remove excess hybridization buffer; three times at 55°C for 30 minutes per wash and at RT for 60 minutes. Slides were then dipped for 30 seconds in 70% (v/v) ethanol/300 mM ammonium acetate, then for 30 seconds in absolute alcohol, air dried and co-exposed with ^12^C microscale standards (Amersham, Piscataway, NJ) to Biomax MS film (Kodak, Rochester, NY) for 7–10 days.

Densitometric analysis of the images was performed using a micro computing imaging device and MCID Elite v.7 software (MCID, Imaging Research Inc., Ontario, Canada). Sections which were anatomically asymmetrical, damaged or in which the hippocampus was not visible were not included in the analysis. Five matching areas for analysis were outlined on the left and right hemispheres of each section: cortex including retrosplenial agranular cortex, primary and secondary motor cortex; hippocampus CA1; CA2; CA3; and dentate gyrus (DG) including polymorph layer DG. Optical density readings were calibrated to the ^12^C microscale standards to give radioactivity quantities in nCi/μg. *SNCA *densitometry was measured in cortex, CA1, CA2, CA3, and dentate gyrus of the treated (right) side of the brain and compared to corresponding regions in the untreated (left) side in two to four sections per animal.

### Immunohistochemistry

5 μm paraffin sections were de-waxed, hydrated and washed in PBS. Endogenous peroxidases were blocked in 0.3% hydrogen peroxide in PBS. To allow epitope unmasking, sections stained for activated microglia with Iba-1 were steamed in distilled water for 30 minutes. Unmasking was not required for α-synuclein. Non-specific sites were blocked with 5% non-fat milk in PBS for 30 minutes. Sections were then incubated for 1 hour at RT with a mouse IgG1 anti-α-synuclein antibody (1:500 dilution, clone 42, BD Biosciences, San Jose, CA) or rabbit Iba-1 antibody (1:1000 dilution, Wako Chemicals USA, Richmond, VA) in 5% non-fat milk. Control slides were set up without primary antibody. Sections were then washed in PBS twice for five minutes and then incubated with anti-mouse biotinylated secondary antibody (Vector Laboratories, Burlingame, CA, USA). After washing, sections were then incubated with Vectastain ABC^® ^reagent in PBS according to the manufacturer's instructions. Signal was visualized with DAB (3',3' diaminobenzidine, Vector Laboratories).

### Statistical analysis

Statistical analysis for *in vitro *studies utilized the t-test to compare groups; Welch's modified t-test was used when variances differed. Numerical variables were summarized with the sample median, 25^th ^percentile, and 75^th ^percentile. The Wilcoxon signed rank sum test was used to test whether the median right/left (R:L) ratio of *SNCA *expression from quantitative RT-PCR differed from 1. The Wilcoxon rank sum test was used to compare qRT-PCR *SNCA *expression between siRNA and control mice groups; it was also used to compare *SNCA *densitometry R:L ratio between groups of mice. Graphical exploration was used to investigate trends in *SNCA *densitometry R:L brain region ratio over different timepoints. Statistical significance was determined at the 5% level.

## Results and discussion

### Screening for active siRNAs *in vitro*

We initially designed nine siRNAs (Mayo 1–9) which are complementary to the *SNCA *transcript in the coding region and the 3'-UTR region (Additional file 1). These siRNAs were screened for their ability to silence the expression of a transiently co-transfected enhanced green fluorescent protein-human *SNCA *fusion construct (pEGFP-NACP) in BE(2)-M17 human neuroblastoma cells. Controls included siRNAMr, specific for the enhanced green fluorescence protein (EGFP) portion of the conjugate, and cells transfected with plasmid DNA in the absence of siRNA. Mayo 2, 7 and 8 were found to produce ≥ 89% silencing (Additional file 2). The controls did not display significant silencing (<43%). In the absence of plasmid co-transfection, quantitative RT-PCR showed that the endogenous *SNCA *mRNA transcript was reduced by 89% for Mayo 2, 52% for Mayo 7 and 67% for Mayo 8 (Additional file 3). In immunoblots endogenous α-synuclein protein showed a 45%, 55% or 53% knockdown for Mayo 2, 7 and 8 respectively (Additional file 3). As opposed to Mayo 7 and Mayo 8, Mayo 2 siRNA diverged from *SNCB *sequence at only four bases; therefore, we demonstrated that Mayo 2 did not silence the closely related endogenous *SNCB *transcript (Additional file 4). To further test the species specificity of Mayo 2, 7 and 8, co-transfection of siRNAs was performed with either human or murine SNCA-pEGFP plasmid. Silencing of human *SNCA *versus murine *SNCA *was 74% and 79% respectively for Mayo2, whereas Mayo 7 and 8 were more human specific (85% human and 47% mouse for Mayo 7 and 73% human and 7% mouse for Mayo8. (Additional file 4)

Since siRNAs can be readily degraded *in vivo*, assays in human serum were performed using modified Mayo 7 and 8 siRNAs (containing either phosphothiorate linkages or 2'-O-methyl substitutions) and enhanced stability was observed (Additional file 5). Modified siRNAs were re-tested for silencing of endogenous SNCA transcript and were found to have maintained their efficacy. A modified version of human specific Mayo8 (Mayo8S2) was selected as the best candidate based on its stability and silencing properties but for *in vivo *testing in mice it was necessary to modify it to complement murine *SNCA *mRNA (Mayo8S2M). *In vitro *testing with either human or murine pEGFP plasmid followed by immunoblot analysis, demonstrated 97% silencing of the murine α-synuclein protein and only 23% of the human α-synuclein protein (Additional file 6).

### Specific *in vivo *knockdown of murine *SNCA*

In order to test the ability of naked siRNA to reduce *SNCA *expression *in vivo*, we identified the hippocampus and the cortex as having the highest expression of *SNCA *in the murine brain (data not shown). We subsequently chose to target siRNA against *SNCA *expression in the hippocampus to decrease variability in our measurements that could be introduced when dissecting out smaller, less defined structures. We delivered Mayo8S2M siRNA against murine *SNCA*, siRNA against luciferase (luc), or PBS into the right CA1 of the hippocampi of wild-type C57BL6 female mice. Infusions were performed on these inbred female mice to reduce variability that can be introduced by combining genders or by having genetically heterogeneous backgrounds. Continuous infusion of the siRNA or PBS solutions was performed over a period of 15 days with Alzet mini pumps connected to cannulae which were surgically implanted into the right CA1. After 15 days, two pumps containing the *SNCA *siRNA and three pumps containing the luc siRNA had disconnected. These mice, represented by open circles, are included in the data analysis in Figure 1. The left CA1 was not injected and was therefore utilized for an untreated control. Hippocampal infusion of the Mayo8S2M siRNA resulted in significant knockdown of *SNCA *when assessed by Taqman quantitative real-time PCR. Normalization was performed against *HPRT *and *GAPDH *as endogenous controls. Quantitative RT-PCR analysis demonstrated that *SNCA *expression was significantly decreased in the right (treated) hippocampus of animals which have received *SNCA *siRNA when compared to the left (untreated) hippocampus (p = 0.037) as demonstrated by the R:L ratio of *SNCA *expression. Additionally, the *SNCA*-specific siRNA reduced *SNCA *expression when compared to luciferase-siRNA (p = 0.004) and PBS (p = 0.036) treated control mice (Figure 1).

Although *SNCA *expression was significantly reduced in the *SNCA *siRNA treated hippocampi, we hypothesized that the efficacy of the siRNA in the brain might be underestimated due to partial diffusion of the siRNA into the contralateral hippocampus. In order to address this issue, we endeavored to examine the distribution of *SNCA *knockdown by *in situ *hybridization so that the extent of *SNCA *reduction could be fully appreciated. Utilizing a mouse *SNCA *specific probe, we determined if the knockdown of *SNCA *expression extended beyond the CA1 cannulation site (Figure 2). *SNCA *knockdown with *SNCA *siRNA was observed an average of 2.67 mm ± 0.57 mm from the cannula tip. While the cannulation tract was evident in the infused animals, in general, little damage nor increase in inflammation (Figure 2C, D, E) was noted at the site of infusion regardless of treatment group, and no animals were removed from the study due to hippocampal damage. Figure 2F shows the hippocampus from the mouse with the most damage at the infusion site. Ratios were calculated for each animal between the treated (right) side (*SNCA *siRNA, luc siRNA, and PBS) and the untreated (left) side and then compared across each of the three groups (Table 1). The least reduction in *SNCA *levels, shown as a reduction in right (R):left (L) ratio, was observed in the cortex (71% between PBS and *SNCA *siRNA treated animals, p = 0.067), likely reflecting the fact that the cortex spans from regions adjacent to the infusion site to regions quite distant from the infusion site and thus less likely to be affected by the siRNA. Significant reductions in *SNCA *levels were observed in the CA1 (66%, p < 0.001), CA2 (59%, p < 0.001), CA3 (77%, p < 0.001), and dentate gyrus (81%, p = 0.001) when *SNCA *siRNA treated animals were compared to PBS treated animals. Similar results were obtained when *SNCA *siRNA treated animals were compared to luc siRNA treated control mice. Reduction in *SNCA *levels was confirmed by immunostaining for murine α-synuclein protein (Figure 2C and 2D). Notably, α-synuclein levels in the cell bodies of the hippocampus were decreased, while α-synuclein in projections from distal regions persisted. Toluidine Blue staining of *in situ *sections showed that decreased *SNCA *levels in mice treated with *SNCA *siRNA were not due to neuronal loss (data not shown).

**Table 1:** Quantitative densitometry of *SNCA in situ *hybridization

	*SNCA densitometry R/L ratio*				
*Brain region*	*PBS (N = 9)*	*Luciferase siRNA (N = 10)*	*SNCA siRNA (N = 11)*	*P-value: SNCA vs. Luciferase*	*P-value: SNCA vs. PBS*

*Cortex*	0.92 (0.82 – 0.98)	0.90 (0.86 – 1.00)	0.27 (0.20 – 0.90)	0.036	0.067
*CA1*	1.02 (0.98 – 1.14)	0.97 (0.93 – 1.06)	0.35 (0.14 – 0.54)	<0.001	<0.001
*CA2*	1.02 (0.93 – 1.05)	1.01 (0.97 – 1.15)	0.42 (0.19 – 0.62)	<0.001	<0.001
*CA3*	1.15 (0.99 – 1.24)	1.09 (1.01 – 1.15)	0.27 (0.12 – 0.71)	<0.001	<0.001
*DG*	1.02 (0.99 – 1.11)	0.99 (0.94 – 1.04)	0.19 (0.10 – 0.68)	0.008	0.001

Using densitometry to determine efficacy of *SNCA *siRNA, *SNCA *expression in the injected (PBS, luciferase siRNA or *SNCA *siRNA) right side was compared to the uninjected left side and compared across treatment groups. P-values were derived from Wilcoxon rank sum test and the sample median (25^th ^percentile – 75^th ^percentile) is given.

### Resilience of *SNCA *knockdown in mice

In order to determine the length of time *SNCA *expression can be repressed following siRNA treatment, we infused *SNCA *siRNA into the right CA1 of four cohorts. Following 15 days infusion, the first cohort (2 W) was harvested as above, while the cannulae were removed from the remaining cohorts which were then allowed to age for 1 week (2 W-1 W), 2 weeks (2 W-2 W), or three weeks (2 W–3 W) post-infusion. One cannula in the 2 W–3 W group was loose at the end of the study. This mouse, represented by a triangle, was included in the data analysis for Figure 3. Following *in situ *for *SNCA*, we observed approximately 60% knockdown in *SNCA *expression in the right CA1 and cortex compared to the uninjected left side (Figure 3) which replicated our previous experiments. Additionally, similar *SNCA *reductions were observed in the right CA2, CA3, and dentate gyrus of mice treated with *SNCA *siRNA (data not shown). *SNCA *levels remained qualitatively reduced 1 week post-infusion in the dentate gyrus (data not shown) and 2 weeks post-infusion in the CA1 (Figure 3A), CA2, CA3 (data not shown), and cortex (Figure 3B). By three weeks post-infusion, *SNCA *levels in the cortex (Figure 3B), CA2, CA3, and dentate gyrus (data not shown) of the siRNA infused side approached control levels. *SNCA *levels in the right CA1 (Figure 3A), the site of injection, remained noticeably reduced when compared to the uninjected control side through three weeks post-infusion. As in the earlier studies, we saw no impact of *SNCA *siRNA on the levels of *SNCB *at any timepoint (Figure 3).

The use of naked siRNAs in the brain has recently been shown be effective against endogenous murine amyloid precursor protein (APP) [25], dopamine transporter (DAT), serotonin transporter (SERT) [26-28], and mutant human huntingtin [29]. The use of RNAi to reduce endogenous α-synuclein expression was demonstrated in SH-SY5Y cells as well as the impact of silencing on dopamine homeostasis and response to mitochondrial toxins *in vitro *[30]. Our study presents the first successful *in vivo *use of stabilized naked siRNA against endogenous *SNCA *and also demonstrates that a close homologue of the target gene, *SNCB*, was not altered by RNA interference with naked siRNA in the brain. This analysis of *SNCB *is particularly important in demonstrating the specificity of the *SNCA *siRNA silencing, and in showing that an increase in *SNCB *expression does not compensate for a reduction in its homologue, *SNCA*. Furthermore, our study also demonstrates that knockdown of *SNCA *lasted for up to three weeks post infusion, in the CA1, and that the effect was not limited to the hippocampus, the immediate site of delivery, but also diffused into the cortex.

While this study focused on *SNCA *knockdown in the hippocampus for technical practicalities, it would be of considerable interest to determine if *SNCA *siRNA would be efficacious in the SN, given its importance in PD. Future work aimed at SN delivery and at silencing *SNCA *in transgenic mouse models for human α-synucleinopathy or toxin models that develop PD like pathology will further enhance our knowledge on the applicability of naked siRNA in the brain and importantly on the suitability of RNA interference *of SNCA *as a future therapeutic target.

## Conclusion

In this study we have characterized naked siRNA duplexes that actively reduce endogenous *SNCA *mRNA *in vitro *and *in vivo*. Following *in vitro *evaluation of nine siRNAs to assess efficacy, specificity and stability, we selected a candidate (Mayo8S2) for *in vivo *testing. After modification to complement the murine sequence (Mayo8S2M), we show that direct infusion of our candidate siRNA into the hippocampi of adult mice resulted in a resilient reduction in the murine *SNCA *transcript level around the site of infusion as well as in more distant sites. This approach will now facilitate a variety of *in vivo *experiments to temporally dissect the impact of *SNCA *up-regulation, aggregation and Lewy-like pathology, in cellular toxicity and neurodegeneration. While considerable work is still needed to optimize delivery, distribution profiles, and stability of the siRNA before this technique could be applied in the clinic, our study provides the foundation for such studies and offers hope that this technique may eventually translate into a neuroprotective therapy for α-synucleinopathies, including PD, DLB and MSA.

## Competing interests

DB, IT, KC, RB, RKP are employees of Alnylam Pharmaceuticals which is developing therapeutics based on RNA interference. DMM reports a provisional application for patent under 37 CFR §1.53 (c) DMM reports a provisional application for patent entitled "Method of Treating Neurodegenerative Disease". Less than $10,000 has been awarded to date. DMM also reports provisional applications for patents entitled "Parkinson's Disease-Related Disease Compositions and Methods" and "Predicting Parkinson's Disease", for which no monies have been awarded to date.

## Authors' contributions

JL designed and managed the *in vivo *studies, and wrote the manuscript. HM performed *in situ *analysis and co-wrote the manuscript. DB led the siRNA development and conceptually contributed to the studies. AH performed the *in vitro *studies, co-wrote the manuscript, and performed the statistical analysis of the *in vitro *data. CZ and ZH performed surgery prep, surgeries, and animal care. SL, AB, SO, KH, CK performed RNA and protein analysis. IT, KC, RB, and RKP participated in the conceptual and technical development of the siRNA. MH and JC performed the statistical analysis of the *in vivo *data. DMM contributed to the conceptual design. MJF designed and managed the *in vitro *studies and significantly edited the manuscript.

## Supplementary Material

Additional file 1 Complementary positions of the nine siRNA reagents (Mayo 1-Mayo 9) in relation to the full length *SNCA *transcript. [NM_000345.2 – longer transcript (isoform NACP140)]. Translation start and stop codons are shown in bold. Click here for file

Additional file 2 Immunoblot analysis of *in vitro *screening of *SNCA *siRNA in BE(2) M17 human neuroblastoma cells. (A) A typical immunoblot showing EGFP and α-tubulin immunoreactivities. Cells were transfected with either pEGFP-C1 (vector) or pEGFP-NACP (α-syn) and one of the Mayo1–9 siRNA reagents or siRNAMr. The three rightmost lanes are no-siRNA controls and an untransfected culture. The conjugated EGFP and α-synuclein product (EGFP/NACP) is retarded by the additional 140 amino acids encoded by the *SNCA *cDNA. (B) Densitometric analysis of combined data from four blots expressed as a fold value of the no-siRNA control according to EGFP:α-tubulin ratio. * p < 0.001, t-test, Welch's modified t-test was used when variances differed. Click here for file

Additional file 3 Silencing of endogenous α-synuclein *in vitro*. (A) qRT-PCR of endogenous *SNCA *transcript from RNA preparations from cells treated with 50 nM siRNAs (Mayo 2, 7, 8, 9, siRNAMr) for 24 h. Each sample was assayed in quadruplicate, and expressed as a fold change from the untransfected control. * p < 0.05 t-test, Welch's modified t-test was used when variances differed. Error bars = SEM. (B) A typical immunoblot of cell extracts following 24 h transfection with 50 nM siRNA. The control is treated with transfection reagent alone. The position of a 16 kDa marker (lysozyme) is indicated. (C) Densitometric analysis of four independent experiments demonstrates significant reduction in the α-synuclein immunoreactivity (IR). * p < 0.05; ** p < 0.01 in t-test, Welch's modified t-test was used when variances differed. Error bars = SEM. Click here for file

Additional file 4 Target specificity of candidate siRNA molecules. (A) RNA preparations from cells treated for 24 h with 50 nM Mayo2 were analyzed by qRT-PCR. Although *SNCA *and *SNCB *diverge by only four bases within the Mayo2 sequence, silencing is specific to *SNCA *only. (B and C) Co-transfection studies in cells demonstrate that Mayo2 is active against both human and mouse *SNCA*, but human specific Mayo7 and Mayo8 do not silence mouse *SNCA *expression. Error bars = SEM, calculated from three independent assays. Click here for file

Additional file 5 Stabilization of siRNAs was achieved by chemical modifications as shown below. Click here for file

Additional file 6 Species specificity of mouse and human *SNCA *siRNA. (A) A typical immunoblot of total protein extracts from cells co-transfected with plasmids conferring expression of EGFP (V; vector) or EGFP-NACP (H = human α-synuclein; M = mouse α-synuclein) alone (control) or with 50 nM of either Mayo8S2 or Mayo 8S2M siRNA. A reprobe of the blot with α-tubulin antibody was used to equalize loading levels. (B) Densitometric analysis of three independent assays demonstrates that silencing of *SNCA *expression by Mayo8S2 is human specific, and by Mayo8S2M is mouse specific. p < 0.01, t-test, Welch's modified t-test was used when variances differed. Error bars = SEM. * Click here for file
