# Supplementary material for: In vivo silencing of alpha-synuclein using naked siRNA
Source: Mol Neurodegener. 2008 Nov 1;3:19. doi: 10.1186/1750-1326-3-19 (PMC2612658; doi:10.1186/1750-1326-3-19)
Supplement: Additional file 1 — Complementary positions of the nine siRNA reagents (Mayo 1-Mayo 9) in relation to the full length SNCA transcript. [NM_000345.2 – longer transcript (isoform NACP140)]. Translation start and stop codons are shown in bold. [file 1750-1326-3-19-S1.doc]

**Met**A spValPheMe

1 ggagtggcca ttcgacgaca gtgtggtgta aaggaattca ttagcc**atg**g atgtattcat

tLysGlyLeu SerLysAlaL ysGluGlyVa lValAlaAla AlaGluLysT hrLysGlnGl

61 gaaaggactt tcaaaggcca aggagggagt tgtggctgct gctgagaaaa ccaaacaggg

yValAlaGlu AlaAlaGlyL ysThrLysGl uGlyValLeu TyrValGlyS erLysThrLy

121 tgtggcagaa gcagcaggaa agacaaaaga gggtgttctc tatgtaggct ccaaaaccaa

sGluGlyVal ValHisGlyV alAlaThrVa lAlaGluLys ThrLysGluG lnValThrAs

181 ggagggagtg gtgcatggtg tggcaacagt ggctgagaag accaaagagc aagtgacaaa

**|--------Mayo 1-------| |-------Ma**

**|--------Mayo 2-------|**

nValGlyGly AlaValValT hrGlyValTh rAlaValAla GlnLysThrV alGluGlyAl

241 tgttggagga gcagtggtga cgggtgtgac agcagtagcc cagaagacag tggagggagc

**yo 4-------| |------Mayo 6------|**

aGlySerIle AlaAlaAlaT hrGlyPheVa lLysLysAsp GlnLeuGlyL ysAsnGluGl

301 agggagcatt gcagcagcca ctggctttgt caaaaaggac cagttgggca agaatgaaga

**|-------Mayo 3--------| |----**

uGlyAlaPro GlnGluGlyI leLeuGluAs pMetProVal AspProAspA snGluAlaTy

361 aggagcccca caggaaggaa ttctggaaga tatgcctgtg gatcctgaca atgaggctta

**---Mayo 5-------| |------Mayo 7------**

rGluMetPro SerGluGluG lyTyrGlnAs pTyrGluPro GluAla

421 tgaaatgcct tctgaggaag ggtatcaaga ctacgaacct gaagcc**taa**g aaatatcttt

**| |------Mayo 8------|**

481 gctcccagtt tcttgagatc tgctgacaga tgttccatcc tgtacaagtg ctcagttcca

541 atgtgcccag tcatgacatt tctcaaagtt tttacagtgt atctcgaagt cttccatcag

601 cagtgattga agtatctgta cctgccccca ctcagcattt cggtgcttcc ctttcactga

661 agtgaataca tggtagcagg gtctttgtgt gctgtggatt ttgtggcttc aatctacgat

721 gttaaaacaa attaaaaaca cctaagtgac taccacttat ttctaaatcc tcactatttt

781 tttgttgctg ttgttcagaa gttgttagtg atttgctatc atatattata agatttttag

841 gtgtctttta atgatactgt ctaagaataa tgacgtattg tgaaatttgt taatatatat

901 aatacttaaa aatatgtgag catgaaacta tgcacctata aatactaaat atgaaatttt

961 accattttgc gatgtgtttt attcacttgt gtttgtatat aaatggtgag aattaaaata

1021 aaacgttatc tcattgcaaa aatattttat ttttatccca tctcacttta ataataaaaa

1081 tcatgcttat aagcaacatg aattaagaac tgacacaaag gacaaaaata taaagttatt

1141 aatagccatt tgaagaagga ggaattttag aagaggtaga gaaaatggaa cattaaccct

1201 acactcggaa ttccctgaag caacactgcc agaagtgtgt tttggtatgc actggttcct

1261 taagtggctg tgattaatta ttgaaagtgg ggtgttgaag accccaacta ctattgtaga

**|-----Mayo**

1321 gtggtctatt tctcccttca atcctgtcaa tgtttgcttt atgtattttg gggaactgtt

**9------|**

1381 gtttgatgtg tatgtgttta taattgttat acatttttaa ttgagccttt tattaacata

1441 tattgttatt tttgtctcga aataattttt tagttaaaat ctattttgtc tgatattggt

1501 gtgaatgctg tacctttctg acaataaata atattcgacc atg
