# Supplementary material for: In vivo silencing of alpha-synuclein using naked siRNA
Source: Mol Neurodegener. 2008 Nov 1;3:19. doi: 10.1186/1750-1326-3-19 (PMC2612658; doi:10.1186/1750-1326-3-19)
Supplement: Additional file 2 — Immunoblot analysis of in vitro screening of SNCA siRNA in BE(2) M17 human neuroblastoma cells. (A) A typical immunoblot showing EGFP and α-tubulin immunoreactivities. Cells were transfected with either pEGFP-C1 (vector) or pEGFP-NACP (α-syn) and one of the Mayo1–9 siRNA reagents or siRNAMr. The three rightmost lanes are no-siRNA controls and an untransfected culture. The conjugated EGFP and α-synuclein product (EGFP/NACP) is retarded by the additional 140 amino acids encoded by the SNCA cDNA. (B) Densitometric analysis of combined data from four blots expressed as a fold value of the no-siRNA control according to EGFP:α-tubulin ratio. * p < 0.001, t-test, Welch's modified t-test was used when variances differed. [file 1750-1326-3-19-S2.doc]

A

###

B
