# Supplementary figures and images for: In vivo silencing of alpha-synuclein using naked siRNA
Source: Mol Neurodegener. 2008 Nov 1;3:19. doi: 10.1186/1750-1326-3-19 (PMC2612658; doi:10.1186/1750-1326-3-19)

###


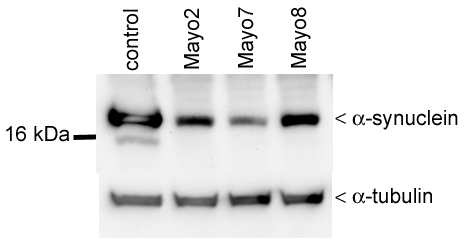


B

A

C

Supplement: Additional file 3 — Silencing of endogenous α-synuclein in vitro. (A) qRT-PCR of endogenous SNCA transcript from RNA preparations from cells treated with 50 nM siRNAs (Mayo 2, 7, 8, 9, siRNAMr) for 24 h. Each sample was assayed in quadruplicate, and expressed as a fold change from the untransfected control. * p < 0.05 t-test, Welch's modified t-test was used when variances differed. Error bars = SEM. (B) A typical immunoblot of cell extracts following 24 h transfection with 50 nM siRNA. The control is treated with transfection reagent alone. The position of a 16 kDa marker (lysozyme) is indicated. (C) Densitometric analysis of four independent experiments demonstrates significant reduction in the α-synuclein immunoreactivity (IR). * p < 0.05; ** p < 0.01 in t-test, Welch's modified t-test was used when variances differed. Error bars = SEM. [file 1750-1326-3-19-S3.doc]

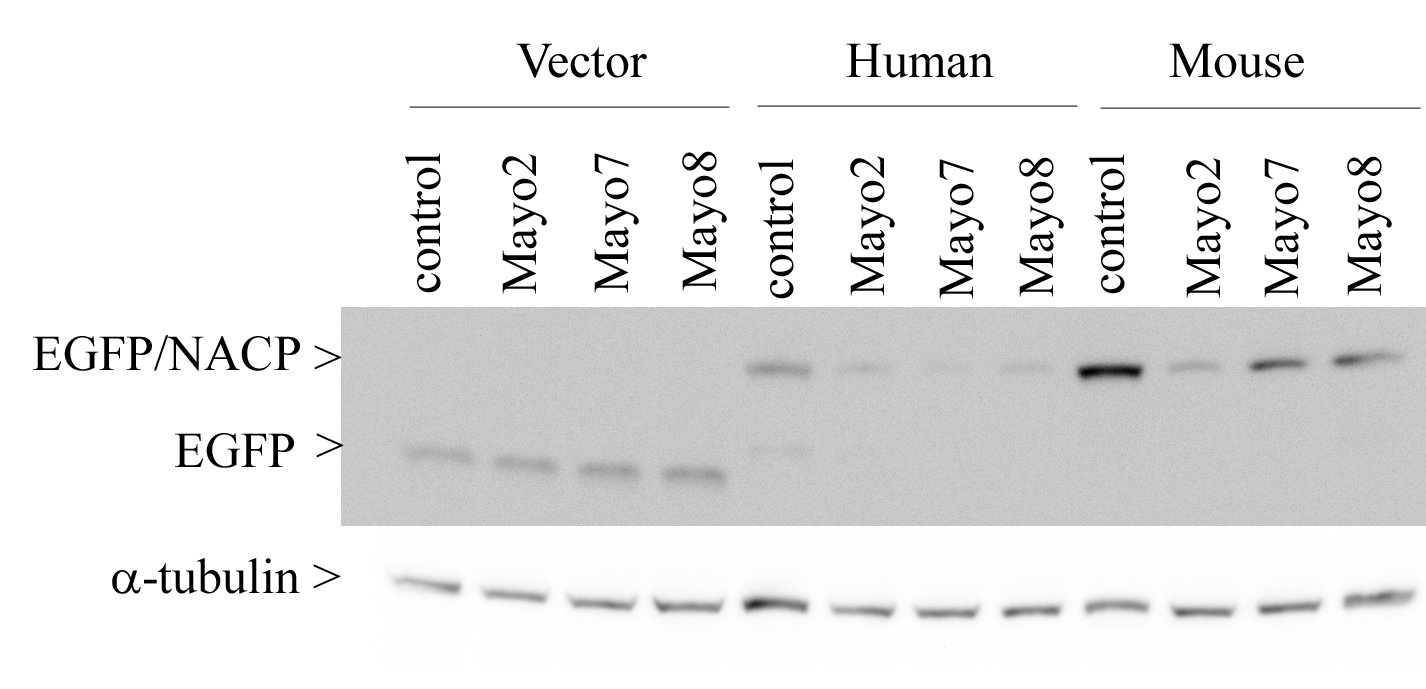


A

B

C

Supplement: Additional file 4 — Target specificity of candidate siRNA molecules. (A) RNA preparations from cells treated for 24 h with 50 nM Mayo2 were analyzed by qRT-PCR. Although SNCA and SNCB diverge by only four bases within the Mayo2 sequence, silencing is specific to SNCA only. (B and C) Co-transfection studies in cells demonstrate that Mayo2 is active against both human and mouse SNCA, but human specific Mayo7 and Mayo8 do not silence mouse SNCA expression. Error bars = SEM, calculated from three independent assays. [file 1750-1326-3-19-S4.doc]

A


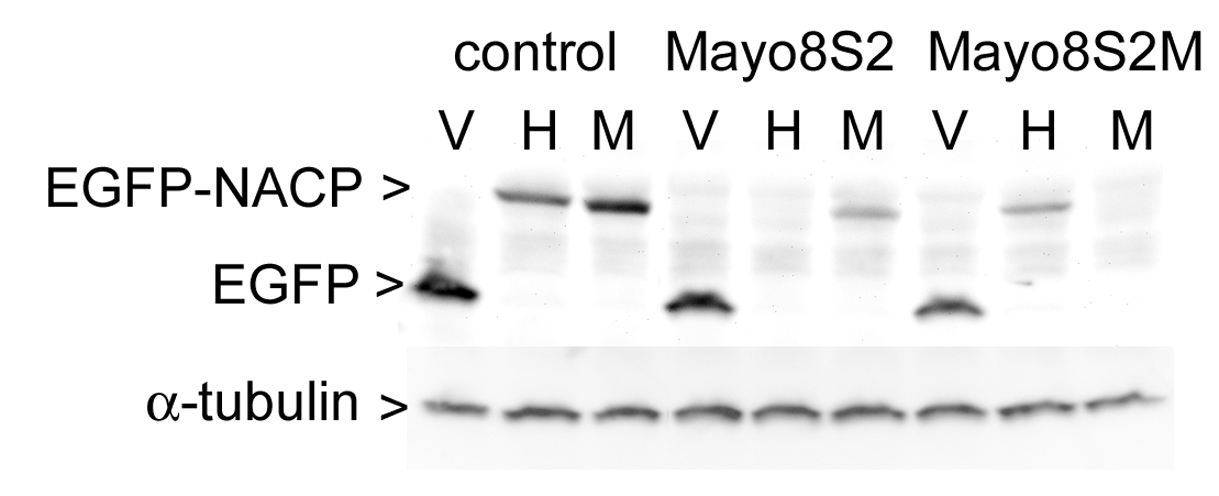


B

Supplement: Additional file 6 — Species specificity of mouse and human SNCA siRNA. (A) A typical immunoblot of total protein extracts from cells co-transfected with plasmids conferring expression of EGFP (V; vector) or EGFP-NACP (H = human α-synuclein; M = mouse α-synuclein) alone (control) or with 50 nM of either Mayo8S2 or Mayo 8S2M siRNA. A reprobe of the blot with α-tubulin antibody was used to equalize loading levels. (B) Densitometric analysis of three independent assays demonstrates that silencing of SNCA expression by Mayo8S2 is human specific, and by Mayo8S2M is mouse specific. p < 0.01, t-test, Welch's modified t-test was used when variances differed. Error bars = SEM. * [file 1750-1326-3-19-S6.doc]
