# Supplementary material for: In vivo silencing of alpha-synuclein using naked siRNA
Source: Mol Neurodegener. 2008 Nov 1;3:19. doi: 10.1186/1750-1326-3-19 (PMC2612658; doi:10.1186/1750-1326-3-19)
Supplement: Supplementary file 4 [file 1750-1326-3-19-S5.doc]

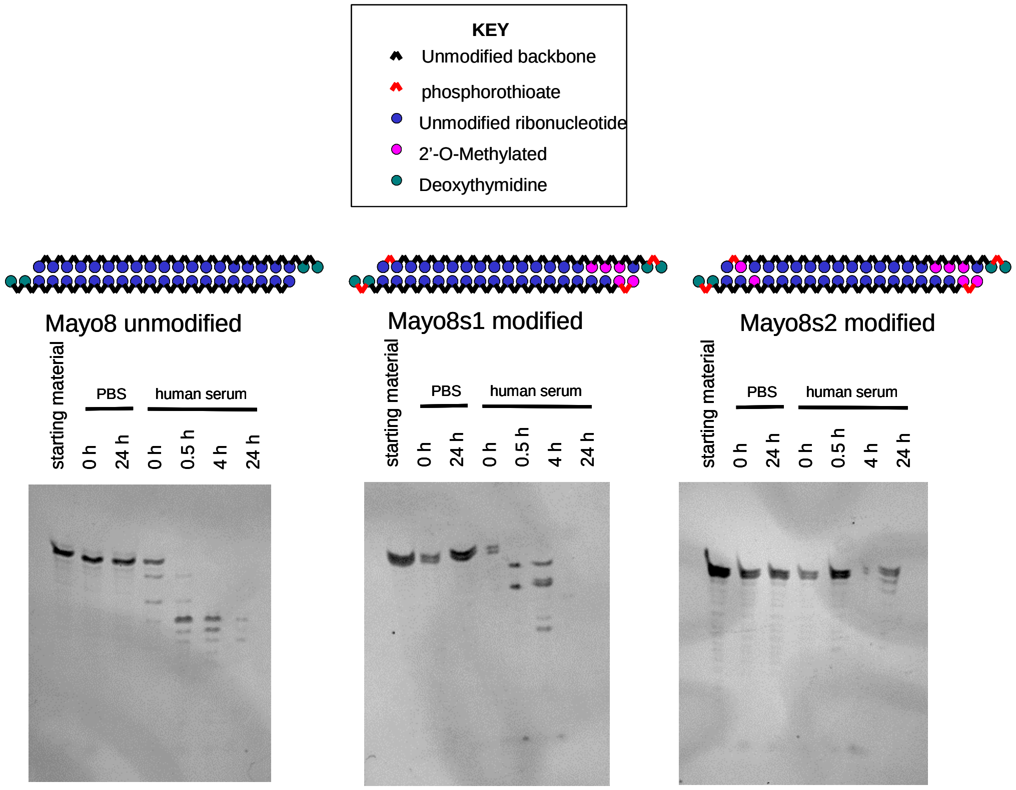
KEY
Unmodified backbone
phosphorothioate
Unmodified ribonucleotide
2'-O-Methylated
Deoxythymidine
Mayo8 unmodified
Mayo8s1 modified
Mayo8s2 modified
starting material
PBS
human serum
0 h
24 h
0 h
0.5 h
4 h
24 h
